# Utilization of machine learning to identify lower extremity biomechanical predictors of rupture in a validated cadaveric model of ACL injury

**DOI:** 10.1038/s41598-026-43183-7

**Published:** 2026-03-09

**Authors:** Parsa Khorrami, Taofeek Braimoh, Dayane Alfenas Reis, Nathaniel A. Bates, Nathan D. Schilaty, John Michael Templeton

**Affiliations:** 1 Bellini College of Artificial Intelligence, Cybersecurity, and Computing, University of South Florida, Tampa, FL 33620 USA; 2 Department of Orthopaedics, The Ohio State University, Columbus, OH 43210 USA; 3 Department of Neurosurgery & Brain Repair, University of South Florida, Tampa, FL 33613 USA; 4 Department of Medical Engineering, University of South Florida, Tampa, FL 33620 USA; 5 Center for Neuromusculoskeletal Research, University of South Florida, Tampa, FL 33620 USA

**Keywords:** Anterior cruciate ligament (ACL), Machine learning, ACL injury prediction, Computational biology and bioinformatics, Diseases, Health care, Medical research, Risk factors

## Abstract

Anterior cruciate ligament (ACL) rupture is a critical concern in sports medicine. This study presents a detailed evaluation of machine learning (ML) techniques for the prediction of anterior cruciate ligament (ACL) injuries - a critical concern in sports medicine that often entails prolonged recovery periods and significant patient burden. Leveraging the transformative potential of artificial intelligence (AI) in medical applications, our work assesses eight distinct ML models (i.e., Support Vector Machines, Decision Tree Classifiers, Random Forest, Stochastic Gradient Descent, Logistic Regression, Gradient Boosting, Ridge Regression, and Linear Discriminant Analysis). Models were trained and tested on four datasets: 53-feature (Binary) ACL Rupture Biomechanical and Demographic (ARBD/BARBD) and 13-feature (Binary) ACL Rupture Wearable (ARW/BARW) that are readily accessible in real-life scenarios. Models were evaluated under a three-class schema ’pre-rupture,’ ’trial prior to rupture,’ ’rupture’ and then reclassified into a binary ’pre-rupture’ vs. ’elevated risk.’ Our analyses reveal that early-phase force metrics, particularly those recorded at 33 milliseconds (e.g., 33ms_Fx and 33ms_Fz) with ARBD and BARBD datasets and initial-contact forces (e.g., IC_Fx, IC_Fz) over demographic variables in ARW and BARW datasets, consistently emerge as significant predictors of injury across multiple and binary models. Notably, across the ARBD and ARW datasets, the ML models achieved accuracies ranging from approximately 79% to 87%, which improved markedly to a range of 92% to 95% when reclassified into a binary classification. These findings underscore the clinical relevance of early dynamic measurements and demonstrate the robustness and generalizability of our approach.

## Introduction

The anterior cruciate ligament (ACL) is a crucial stabilizer of the knee joint, connecting the femur to the tibia. It plays a vital role in maintaining knee stability during various movements, particularly in athletic activities^1^. ACL injuries are prevalent, with approximately 300,000 tears occurring annually in the United States^2^. These injuries typically occur during sports activities, highlighting a vital need for real-time detection and risk identification. Unfortunately, ACL tears often have profound long-term consequences. Regardless of whether the injury is managed via conservative treatment or surgical reconstruction, patients frequently experience abnormal sports function and altered biomechanics. These persistent deficits contribute to unfavorable long-term outcomes, including a significantly increased risk of early-onset knee osteoarthritis within 10 years post-injury^2,3^. Given these risks, identifying injury risk factors and preventing ACL rupture is essential.

Current standards for the clinical diagnosis of ACL rupture are based on a stepwise combination of focused history, physical examination, and medical imaging. While knee arthrography is a gold standard, it is rarely implemented due to its invasive nature^4^. Instead, systematic reviews identify Lachman, anterior-drawer, and pivot-shift tests as the cornerstones of clinical assessment^5^. The Lachman test is approximately 81% sensitive to detect complete tears^6^, while the pivot-shift test exhibits strong specificity. Newer data also support the lever-sign test as a valuable adjunct to rule out injury^7^. When confirmation or surgical planning is required, Magnetic Resonance Imaging (MRI) serves as the imaging gold standard, detecting ruptures with roughly 87% sensitivity and 93% specificity^8^. Following diagnosis, ACL reconstruction (ACLR) is the most commonly used treatment, with estimates of 400,000 procedures performed annually in the US^4^. Modern ACLR focuses on replacing the torn ligament with a biologic graft—such as quadriceps tendon or semitendinosus autografts—to optimize biomechanical stability. Contemporary techniques, including all-inside approaches, utilize precise tunnel creation to reduce trauma and enhance rehabilitation potential^9^. However, these conventional methods are inherently retrospective and reactive.

Artificial Intelligence (AI) has emerged as a transformative tool in musculoskeletal medicine, offering promising avenues to shift from reactive treatment to prospective risk assessment^10^. In the realm of musculoskeletal injuries, particularly ACL injuries, AI offers promising avenues to enhance early detection and relative risk assessment.

Jauhiainen et al.^11^ analyzed an extensive screening battery of elite female athletes and demonstrated that ML models could achieve statistically significant predictive performance, though their sensitivity was limited by the narrow range of biomechanical markers considered. Similarly, Girard et al.^12^ employed decision tree algorithms to classify knee injury status based on return-to-activity criteria, highlighting the feasibility of using simple yet interpretable models for injury risk assessment. In another study, Minamoto et al.^13^ applied deep convolutional neural networks to detect ACL tears with high sensitivity and specificity, although their focus was more on post-injury detection rather than proactive risk prediction. These earlier works laid important groundwork by confirming the potential of ML in this domain, yet they often relied on single-model approaches and a limited set of traditional biomechanical features such as joint angles, joint moments, hop performance metrics, strength/laxity measures, and basic spatiotemporal variables.

Multiplanar loading markedly elevates knee-ligament strain, with knee abduction moment (KAM), anterior tibial translation (ATT), and internal tibial rotation (ITR) emerging as the primary key biomechanical predictors of ACL injury risk. A study by Bates et al. (2019)^14^ demonstrated that multiplanar loading incorporating sagittal, frontal, and transverse plane forces significantly increases strain on the ACL and MCL, with knee abduction moment (KAM) having the greatest impact on ACL strain, followed by anterior tibial translation (ATT). These findings highlight the complexity of knee mechanics during dynamic movements, where KAM, ATT, and internal tibial rotation (ITR) are critical biomechanical factors influencing ligament strain, particularly in the context of ACL injuries. KAM increases strain on both the ACL and MCL, especially under multiplanar loading, due to the added stress from abduction forces resisting lateral loads^15^. ATT directly elevates ACL load during deceleration tasks such as landing or pivoting, which are common in sports, while ITR adds torsional stress that further amplifies ACL strain, especially when combined with KAM. The interplay of these forces, particularly under simulated impact conditions^14^, creates a high-risk environment for injury. Furthermore, machine learning models have validated the predictive importance of KAM, ATT, and ITR, underscoring their relevance in injury prevention and rehabilitation strategies^16^.

Monitoring and predicting injury risk in real time is becoming increasingly important as machine learning advances. ML models utilize biomechanical data to predict injury likelihood with increasing accuracy, as seen in deep convolutional neural networks that have been used to detect ACL tears with high sensitivity and specificity^13^. Despite advances in biomechanical modeling and ML, there is a need for real-time data collection and feedback mechanisms that can assist athletes during dynamic movements, preventing injury before it occurs. This gap highlights the importance of further research focused on integrating real-time monitoring with predictive modeling to enhance both injury prevention and rehabilitation strategies^17^.

This study evaluated eight distinct ML models — Support Vector Machines, Decision Tree, Random Forest, Stochastic Gradient Descent, Logistic Regression, Gradient Boosting, Ridge Regression, and Linear Discriminant Analysis — to rigorously extract and rank the most influential biomechanical predictors of ACL injuries in a validated laboratory injury model. We used two primary datasets: (1) a laboratory controlled dataset constructed from 53 biomechanical and demographic features, and (2) a reduced, wearable-compatible dataset containing 13 features that can be realistically obtained from non-invasive sensors in real-world scenarios. In both cases, we explored two classification schemes: a three-class label classification (i.e., pre-rupture, trial immediately prior to rupture, and rupture) and a binary label (i.e., pre-rupture vs. elevated risk), yielding four distinct datasets: (1) ACL Rupture Biomechanical and Demographic (ARBD), (2) Binary ACL Rupture Biomechanical and Demographic (BARBD), (3) ACL Rupture Wearable (ARW), and (4) Binary ACL Rupture Wearable (BARW). By comparing ML models performance across these datasets, we sought to determine which biomechanical variables most consistently emerge as predictors of ACL failure in both laboratory settings and data that can be gathered in practical, real-life scenarios. Findings of this study could lay the groundwork for future studies to integrate real-time, wearable-based monitoring systems into athletic and military settings, enabling proactive interventions—such as neuromuscular training cues or load-management feedback—to mitigate ACL injury risk.

## Methodology

### Data collection and labeling

The dataset includes a total of 51 cadaveric specimens (25 males, 26 females) from a controlled laboratory setting using sensor technology. Each specimen had 192 features that were classified into 4 classes (0 = pre-rupture, 1 = trial prior to rupture, 2 = rupture, and 3 = post-rupture). Specimens with prior knee trauma, surgery, or age > 53 years were excluded, with an average age of 41 years old (minimum 22 and maximum 52 years old) and an average mass of 83.9 kg (a range of 45.4–139.4 kg). All methods were carried out in accordance with relevant guidelines and regulations. Ethical approval for this study was waived by the Mayo Clinic Institutional Review Board (application 15–005819) as non-human subject research. The individuals did consent to the use of their body for scientific purposes via Anatomical Gift Registry (AGR). The data collection protocol is described in literature^18^ where a custom mechanical impact simulator was utilized to replicate *in vivo*^19^ landing mechanics by applying KAM, ATT, ITR, quadriceps force, and hamstrings force via pneumatic actuators then delivering an impulse force equivalent to *in vivo* vertical ground reaction forces to the sole of the foot. Load magnitudes were derived from motion analysis of 67 healthy athletes (23.2 ± 3.9 years; 73.3 ± 13.4 kg), with kinetic data collected during drop vertical jumps. This motion analysis was collected as part of an *in vivo* 3D motion capture in which the length of the limb is part of the calculation to obtain moments. As such, for respective simulations, the provision of applied moments to the knee (i.e., created by a force over a distance), does not need to input the height of the specimen. Mechanical impact simulator sensors included a 6-axis load cell for joint forces/moments (Omega 160, ATI Industrial Automation, Cary, NC, USA), uniaxial load cells for vertical ground reaction force (Interface, Scottsdale, AZ, USA), and custom microminiature differential variable strain transducers (DVRTs, LORD MicroStrain, Williston, VT, USA) implanted on the anteromedial ACL bundle and midsubstance of the medial collateral ligament (MCL) to measure ligament strain. Sub-failure testing involved 46 randomized impacts (34.0 kg sled weight) with KAM, ATT, and ITR loads set to 0–100^th^ percentiles of *in vivo* data. For any specimen that completed sub-failure testing, failure testing increased the weight sled mass to 0.5 $\times$ bodyweight and started simulations at the 100^th^ percentile of loading and incrementally increased KAM, ATS, and ITR loads in 20% increments per trial until ACL rupture or catastrophic knee damage was achieved^20^. A board-certified orthopedic surgeon assessed ligament integrity pre-/post-testing via clinical exams (Lachman’s test, Pivot-Shift test, arthroscopy) and documented injury patterns.

Labeling criteria focused on ACL rupture confirmation and injury characteristics. Data were labeled as “failure” or “intact” based on DVRT-derived strain patterns (loss of elastic rebound indicated rupture) and surgeon-validated clinical grades (e.g., grade 3 Lachman’s test). Features included peak ACL/MCL strains, forces/moments from load cells, and applied external loads (KAM, ATS, ITR). Strain thresholds were calculated using DVRT voltage inflection points, while clinical exams provided categorical labels (e.g., femoral avulsion, midsubstance tear). All data were synchronized via electronic triggers and sampled at 10 kHz. Post-processing involved extracting peak strain values preceding failure and correlating sensor outputs with surgeon assessments to ensure clinical relevance.

### Data preprocessing

In this study, data was collected from cadaveric specimens. Occasional data collection failures resulted in missing values; consequently, samples with at least one missing value were excluded from the dataset to maintain data integrity. To align the study with real-time monitoring applications, the number of classes was reduced from four classes to three (i.e., “pre-rupture”, “trial prior to rupture”, “rupture” classes). This reduction involved the elimination of all samples classified as class 3 or “post-rupture” class, which contained data recorded post rupture. Additionally, all rows and columns containing $\textrm{NaN}$ data were removed. As a result of this process, certain features and samples with missing data were excluded. The remaining feature set was reviewed in collaboration with clinical experts to identify a shortlist of clinically monitorable features. Furthermore, sensor data collected at a 100 ms sampling rate was excluded from the final feature list, as it was obtained too long after the ACL rupture to be considered relevant. This iterative process reduced the feature set from 192 to 53, as these 53 features were specifically related to knee response, were captured by sensors, and demographic features.

Due to the experimental design, a far greater number of “pre-rupture” class samples were contributed by each subject compared to “trial prior to rupture” class and “rupture” class. In the experimental protocol, each leg was placed in a machine that exerted progressive pressure on the knee under various external loads, KAM, ATT, and ITR. The size of the loads was progressively escalated up to the point of ligament rupture. The tear time was labeled as “rupture” class, and the sample immediately preceding it was labeled as “trial prior to rupture” class. Consequently, the dataset is extremely imbalanced, with most samples being “pre-rupture” class.

After executing the aforementioned preprocessing steps, of the original 51 subjects, 9 were eliminated due to corrupted data or missing values, resulting in a final dataset of 42 subjects (20 male, 22 female). A single sample from each subject was contributed to “trial prior to rupture” class and “rupture” class. To counter the challenge posed by both the limited number of samples and the imbalance of the classes, data augmentation was employed to increase the representation of “trial prior to rupture” class and “rupture” class. This was achieved by altering the original data’s sampling rate^21^, which increased the number of samples per specimen for these two classes from one to ten. Starting from the original 10 kHz sampling rate, more samples were generated by decreasing the sampling rate to 9 kHz, 8 kHz, 7 kHz, 6 kHz, 5 kHz, 4 kHz, 3 kHz, 2 kHz, and 1 kHz. This process effectively augmented the size of the samples in “trial prior to rupture” class and “rupture” class. After augmenting data for “trial prior to rupture” class and “rupture” class, the total number of samples increased from 1721 to 2237, with a list of 53 features. This data augmentation approach significantly improved the accuracy of the machine learning models.

### Dataset

From the same experimental corpus introduced in the “Data Collection and Labeling” section and followed preprocessing steps as described in the “Data Preprocessing” section, four datasets have originated. From that preprocessed dataset we created two feature variants and two class variants so that each dataset highlights a different aspect of the problem (e.g., full biomechanical features vs. wearable-feasible features; three-class vs. binary risk labeling). The datasets utilized are: (1) ACL Rupture Biomechanical and Demographic (ARBD), (2) Binary ACL Rupture Biomechanical and Demographic (BARBD), (3) ACL Rupture Wearable (ARW), and (4) Binary ACL Rupture Wearable (BARW). Appendix Table 8 lists the features of these four datasets. All 53 sensor-based features are included in ARBD and BARBD datasets, and the subset of features with an asterisk are in the ARW and BARW datasets.

The ARBD dataset contains data collected in a laboratory setting with “pre-rupture”, “trial prior to rupture”, “rupture” classes, where the ACL rupture process was experimentally induced. In this setup, a weight was dropped onto the participant’s knee, applying increasing pressure until ACL rupture occurred^18^. The weight was gradually increased to simulate progressive loading conditions. This allowed researchers to observe the knee’s response over time, labeling the data into three classes. However, due to each specimen’s uniqueness and the complexity of observing the exact rupture moment under experimental conditions, distinguishing between “trial prior to rupture” class and “rupture” class proved challenging. For some specimens, there was a gray area where it was unclear whether the sample should be labeled as a rupture or not. To address this, the BARBD dataset was defined. It closely mirrors ARBD but introduces a binary classification scheme by merging “trial prior to rupture” class and “rupture” class into a single category, labeled “elevated risk”. As a result, the new dataset includes two classes: ”pre-rupture” and “elevated risk”. This modification aims to reduce the uncertainty associated with borderline cases and improve classification consistency.

The ARW dataset was introduced to reflect the type of features that can realistically be collected using wearable sensors in real-life scenarios, as determined by biomechanical experts. Although the number of samples and classes remained the same as in ARBD (2,237), the feature set was reduced from 53 to 13 to align with the practical limitations of wearable device data collection. Similarly to the idea of BARBD, the BARW dataset was derived from ARW by combining “trial prior to rupture” class and “rupture” class into a single “elevated risk” category, resulting in a binary-labeled dataset suitable for real-world, wearable-based ACL rupture risk assessment.

### Statistical analysis

The 53 features of the processed dataset were analyzed for statistical differences across the three classes using ANOVA. Subsequently, any significant features were subjected to a t-test to test for significant difference between pairs of each class (i.e “pre-rupture” class vs “trial prior to rupture” class, “pre-rupture” class vs “rupture” class, and “trial prior to rupture” class vs “rupture” class).

### Machine learning models

To determine the most significant features for classification, the following ML models were employed: Support Vector Machine (SVM), Decision Tree classifier, Random Forest classifier, Stochastic Gradient Descent (SGD), Logistic Regression, Gradient Boosting classifier, Ridge Regression, and Linear Discriminant Analysis (LDA). Each model was trained on the dataset to evaluate its effectiveness in identifying key predictive features. Each model’s hyperparameters were fine-tuned within the ranges specified in Table 1 using a grid search strategy, to identify the optimal configuration based on the dataset. The selected models have been previously applied in similar studies related to ACL injury prediction, as referenced below.

Python version 3 was utilized for data processing, modeling, and visualization in this study. The analysis employed several Python libraries, including SciPy for scientific computing, Scikit-learn (sklearn) for machine learning model implementation, and Plotly for interactive data visualization.

**Table 1:** Hyperparameter spaces used for the machine learning models.

Model	Hyperparameter spaces
Support Vector Machine	**C**: [0.001, 0.01, 0.1, 1, 2]; **kernel**: [’linear’]; **degree**: [2, 3, 4, 5]; **gamma**: [0.0001, 0.001, 0.01, 0.1, 1, ’scale’, ’auto’]; **coef0**: [−1, 0, 1, 2, 3]
Decision Tree	**criterion**: [’gini’, ’entropy’]; **max_depth**: [5, 10, 15, 20, None]; **min_samples_split**: [2, 5, 10]; **min_samples_leaf**: [1, 2, 5, 10]
Random Forest	**n_estimators**: [50, 100, 200]; **max_depth**: [None, 10, 20, 30]; **min_samples_split**: [2, 5, 10]; **min_samples_leaf**: [1, 2, 5]
Stochastic Gradient Descent	**loss**: [’log_loss’, ’hinge’, ’modified_huber’]; **alpha**: [0.0001, 0.001, 0.01, 0.1]; **max_iter**: [100, 500, 1000, 2000]; **learning_rate**: [’constant’, ’optimal’, ’invscaling’, ’adaptive’]; **eta0**: [0.001, 0.01, 0.1]
Logistic Regression	**penalty**: [’l1’, ’l2’, ’elasticnet’, ’none’]; **solver**: [’lbfgs’, ’saga’, ’liblinear’]; **C**: [0.001, 0.01, 0.1, 1, 10]; **max_iter**: [1000, 2000, 5000]; **l1_ratio**: [0.1, 0.5, 0.9]; **tol**: [1e-3, 1e-4]
Gradient Boosting	**n_estimators**: [50, 100, 150, 200]; **learning_rate**: [0.001, 0.01, 0.1, 0.2]; **max_depth**: [2, 3, 5, 7]; **min_samples_split**: [2, 5, 10]
Ridge Regression	**alpha**: [0.1, 0.5, 1, 4, 5, 8, 10, 12, 15, 20, 30, 40, 50, 60, 70, 80, 90, 100, 110, 130, 150, 200, 250, 260, 270, 280, 300, 320, 350, 380, 400, 420, 500, 600]
Linear Discriminant Analysis	**n_components**: 2; **solver**: [‘svd’, ‘eigen’]]; **shrinkage**: [0.1, 1, ’auto’]

#### Support vector machine

SVM is a supervised learning algorithm that constructs a hyperplane in a high-dimensional space to classify data points into distinct categories. In a study by Jauhiainen et al., a linear SVM was utilized to predict ACL injuries among 791 female elite handball and soccer players, achieving a mean area under the receiver operating characteristic curve (AUC-ROC) of 0.63, indicating statistically significant predictive ability^11^.

#### Decision tree classifier

A Decision Tree Classifier is a non-parametric model that recursively partitions data based on feature values to create a tree-like structure for decision-making. Rugg et al. implemented a decision tree learning algorithm to classify knee injury status using return-to-activity criteria, demonstrating its potential in evaluating functional capacity related to injury status in adolescent females^12^.

#### Random forest classifier

Random Forest is an ensemble learning method that constructs multiple decision trees during training and outputs the mode of their predictions for classification tasks. In the study by Jauhiainen et al., Random Forest was among the classifiers used to predict ACL injuries, although the linear SVM yielded the highest mean AUC-ROC^11^.

#### Stochastic gradient descent

SGD is an iterative optimization algorithm that updates model parameters based on the gradient of the loss function with respect to a single training example, facilitating efficient learning in large-scale datasets. While specific applications of SGD in ACL injury prediction were not identified in the reviewed literature, its utility in training linear classifiers makes it a viable candidate for such studies.

#### Logistic regression

Logistic Regression is a statistical model that estimates the probability of a binary outcome based on one or more predictor variables. Jauhiainen et al. included logistic regression in their comparative analysis of ML methods for ACL injury prediction, reinforcing its relevance in this context^11^.

#### Gradient boosting classifier

Gradient Boosting is an ensemble technique that builds models sequentially, each correcting the errors of its predecessor, to enhance predictive accuracy. Leckey et al. reviewed various ML approaches, noting that tree-based solutions like Gradient Boosting provided high statistical predictive performance in injury risk prediction^22^.

#### Ridge regression

Ridge Regression is a linear regression technique that incorporates L2 regularization to prevent overfitting by penalizing large coefficients. Although specific studies applying Ridge Regression to ACL injury prediction were not found in the current literature, its capacity to handle multicollinearity suggests potential applicability in such analyses.

#### Linear discriminant analysis

LDA is a multi-class classification and dimensionality reduction that maximizes class separability using optimal linear combinations of features. In its applications for ACL injury prediction, a study conducted by Schilaty et al. demonstrated that an LDA model incorporating 15 biomechanical factors achieved an accuracy of 94.1% in predicting specific knee injury outcomes, highlighting LDA’s efficacy in this domain^23^.

### Model evaluation

The ML models were evaluated for their ability to classify three classes: “pre-rupture”, “trial prior to rupture”, and “rupture” for ARBD and ARW datasets, and two classes: ”pre-rupture” and “elevated risk” for BARBD and BARW datasets. To assess model performance, the train and test sets were partitioned based on specimens identity. The dataset was divided such that 34 specimens formed the training set, while 8 specimens constituted the test set. This specimens-wise splitting method prevents data leakage and ensures that the models generalize well to unseen subjects.

The primary objective was to based on each dataset identify the most influential features for classification tasks. Predictive performance metrics (e.g., accuracy, F1-score) are utilized here as a validation metric for feature selection, ensuring that the biomechanical interpretations are grounded in statistically rigorous model performance. As feature rankings can vary across different machine learning algorithms, the classification performance of each model has been reported to enable a more comprehensive and informed comparison. First, the ML models have been trained on the training set. Next, trained models’ performance have been reported on the test set with Accuracy, Macro Average F1-Score, Macro Average Precision, and Macro Average Recall^24^. The accuracy reflects the performance of each model. However, due to the imbalance in the dataset, we report the Macro average of F1-Score, Precision, and Recall. For example, Ave F1-Score is the average F1-Score across all classes in multiclass classification, giving each class equal weight regardless of size or sample count. In datasets with imbalanced class distributions, standard performance metrics may favor the majority class. Macro-averaged metrics mitigate this bias by calculating precision, recall, and F1 scores for each class independently and then averaging them. This method ensures equal representation of all classes, offering a more balanced evaluation of model performance.

While model performance was evaluated using a strict specimen-wise train/test split to prevent data leakage, we utilized a different strategy for the final feature ranking. Due to the inherent sample size limitations of cadaveric studies, feature importance scores derived from a single training fold is not optimal, as it excludes data from the test set. Therefore, to derive the most stable global approximation of clinical feature list, we retrained the models from scratch on the entire dataset. This approach allows the models to learn from the complete distribution of biomechanical patterns, ensuring that the reported feature rankings represent the most stable global approximation of injury predictors.

#### Evaluation metrics

Accuracy Accuracy (Acc) is a fundamental metric that measures the proportion of correctly classified instances among all instances:

1 $$\begin{aligned} \text {Acc} = \frac{TP + TN}{TP + TN + FP + FN} \end{aligned}$$

While accuracy provides a general measure of performance, it is essential to report precision, recall, and the F1 score as well. Precision assesses the correctness of positive predictions, recall evaluates the model’s ability to identify all relevant instances, and the F1 score offers a balanced measure of both, particularly useful in imbalanced datasets.

Macro Averaged Metrics Since this study involves multiclass classification, we calculate the macro-average of Precision ($P_{ma}$), Recall ($R_{ma}$), and F1-Score ($F1_{ma}$) to treat all classes equally:

2 $$\begin{aligned} P_{ma} = \frac{1}{N} \sum _{i=1}^{N} \frac{TP_i}{TP_i + FP_i} \end{aligned}$$

3 $$\begin{aligned} R_{ma} = \frac{1}{N} \sum _{i=1}^{N} \frac{TP_i}{TP_i + FN_i} \end{aligned}$$

4 $$\begin{aligned} F1_{ma} = \frac{1}{N} \sum _{i=1}^{N} 2 \times \frac{\left( \frac{TP_i}{TP_i + FP_i}\right) \times \left( \frac{TP_i}{TP_i + FN_i}\right) }{\left( \frac{TP_i}{TP_i + FP_i}\right) + \left( \frac{TP_i}{TP_i + FN_i}\right) } \end{aligned}$$

where $N$ represents the number of classes.

## Results

In this section, we begin by presenting the outcomes of the statistical analyses to identify significant patterns and differences within the datasets. These results provide important context for understanding the predictive modeling that follows. Subsequently, for each of the four datasets, the performance of the machine learning models is evaluated in terms of classification accuracy and other relevant evaluation metrics. In addition, we provide a ranked list of features based on their contribution to model performance, highlighting the most influential variables across the different datasets.

### Statistical analyses

An overview of the results obtained through ANOVA showed that of the 53 features, 3 features did not show any statistical significance. These nonsignificant features include, IC_Mz (N-m), 33ms_ACL Poly (mm), and 67ms_ACL Poly (mm) which represent Moment in Z at initial contact, ACL displacement (mm) at 33 ms after initial contact calculated with polynomial equation, and ACL displacement (mm) at 67 ms after initial contact calculated with polynomial equation respectively. The results of the subsequent t-test for the remaining 50 significant features are as follows, 48 (90.56%) of features showed substantial difference between “pre-rupture” class and “trial prior to rupture” class, 46 (86.79%) of features showed significant difference between “pre-rupture” class and “rupture” class, and 19 (35.84%) features showed significant difference between “trial prior to rupture” class and “rupture” class.

### ARBD dataset

**Fig. 1:**
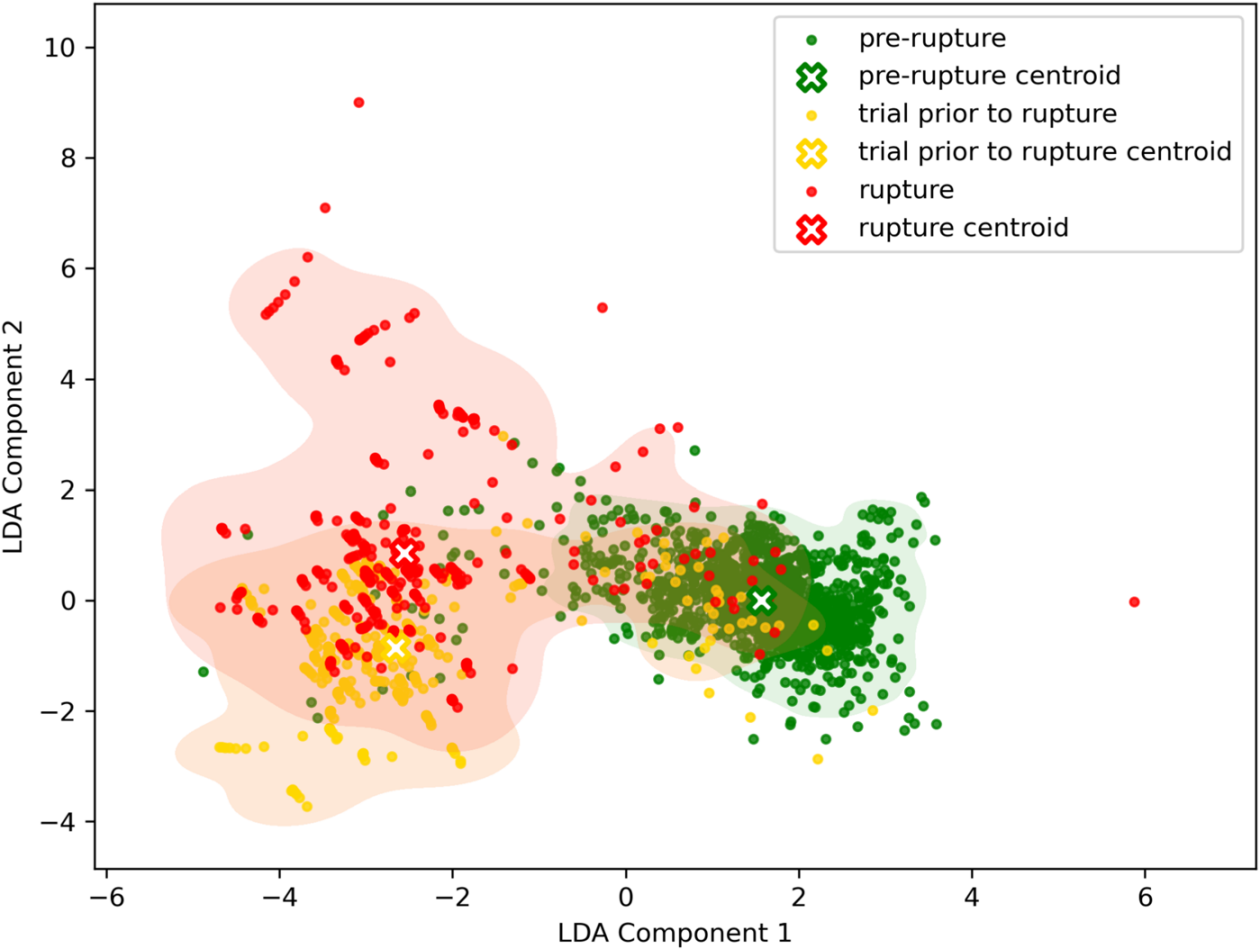
LDA of ARBD dataset.

To identify the ranked feature list, it is essential to first evaluate the performance of each model. The high classification performance of a model indicates the reliability of its proposed ranked feature list. Table 2 presents a summary of the accuracy scores, Ave F1-Score, Ave Precision, and Ave Recall achieved by each model in the test set. Among all models, LDA demonstrated the highest performance, achieving an accuracy of 86.9% and an F1-score of 79.7%. In contrast, other models, such as the decision tree, exhibited lower accuracy and F1-scores. Figure 1 shows the plot obtained between the components derived from the linear combinations of all 53 features by LDA on the ARBD dataset. Samples from each class are represented using three distinct colors: green for the “pre-rupture” class, yellow for the “trial prior to rupture” class, and red for the “rupture” class. While a perfect clustering pattern is not observed in the figure, it is evident that the majority of samples belonging to the “trial prior to rupture” and “rupture” classes are concentrated on the left side of the plot. In contrast, samples from the “pre-rupture” class tend to be distributed toward the right side of the plot.

**Table 2:** Classification performance of ML models on ARBD and BARBD dataset.

Dataset	Model	Acc (%)	$F1_{ma}$(%)	$P_{ma}$(%)	$R_{ma}$(%)
ARBD	LDA	86.9	79.7	79.4	80.2
	Ridge Regression	85.6	78.0	78.3	77.7
	Logistic Regression	84.5	76.9	76.6	77.1
	Gradient Boosting	84.5	74.1	76.3	74.1
	SVM	81.9	75.6	76.5	75.5
	Random Forest	80.6	66.0	66.7	66.3
	Decision Tree	80.0	65.1	66.2	65.7
	SGD	79.7	71.5	71.5	71.5
BARBD	LDA	93.9	93.3	93.2	93.4
	Ridge Regression	93.9	93.3	93.2	93.4
	Logistic Regression	95.0	94.5	94.6	94.3
	Gradient Boosting	94.6	94.0	93.9	94.1
	SVM	93.0	92.4	92.0	92.9
	Random Forest	95.0	94.5	94.5	94.4
	Decision Tree	94.8	94.2	94.4	94.1
	SGD	93.5	92.9	92.5	93.2

ARBD: ACL Rupture Biomechanical and Demographic.

BARBD: Binary ACL Rupture Biomechanical and Demographic.

SVM: Support Vector Machine.

LDA: Linear Discriminant Analysis.

SGD: Stochastic Gradient Descent.

Table 3 presents the frequency with which features appeared in the top-ten ranked feature lists of each model. Features such as MAX_Mx (N-m), appearing in the top-ten feature list for 75% of the models, were identified as consistently important based on the majority occurrence.

Appendix Table 9 lists the top-five ranked feature list of each model, representing the relative contribution of each feature to the classification task. Features like 33ms_Fx (N) and MAX_Mx (N-m) appear frequently across different models, indicating their consistent and substantial importance in distinguishing between classes.

**Table 3:** Frequency of Features Appearing in the Top 10 Lists Across Models based on ARBD dataset.

Feature	Frequency
MAX_Mx (N-m)	6
33ms_Fx (N)	6
MAX_Fy (N)	5
33ms_Fz (N)	5
MIN_Fx (N)	4
MIN_Mx (N-m)	4
IC_Fy (N)	4
MAX_Mz (N-m)	3
MAX_ACL Linear Strain (%)	3
IC_Fx (N)	2
MIN_ACL Linear (mm)	2
MAX_My (N-m)	2
Height (cm)	2
IC_ACL Linear (mm)	2
33ms_My (N-m)	2
MIN_Fy (N)	2
MIN_ACL Linear Strain (%)	2
MAX_Fx (N)	2
MIN_Fz (N)	2
Sex	2
33ms_Fy (N)	1
IC_ACL Poly (mm)	1
IC_ACL Linear Strain (%)	1
67ms_My (N-m)	1
Weight (lbs)	1
33ms_ACL Linear Strain (%)	1
MIN_ACL Poly (mm)	1
67ms_ACL Poly (mm)	1
33ms_Mz (N-m)	1
67ms_Fz (N)	1
IC_Mx (N-m)	1
MAX_ACL Linear (mm)	1
MIN_Mz (N-m)	1
67ms_ACL Linear (mm)	1
33ms_ACL Linear (mm)	1
IC_My (N-m)	1
IC_Mz (N-m)	1

### BARBD dataset

This dataset has two classes, “pre-rupture” and “elevated risk”, which is similar to ARBD dataset, but classes one and two were merged into one class as the “elevated risk” class. Table 2 summarizes the performance of the ML model in the test set, random forest and logistic regression share the best performance with 95% accuracy and 94.5% F1-Score. Based on the LDA algorithm, the number of LDA components is at most equal to the min (number of classes - 1, number of features). Due to the binary nature of the classes, an LDA plot could not be generated because the LDA component had only one dimension.

Table 4 lists the frequency of the top-ten features across all ML models. 33ms_Fx (N) is the most repeated feature in the top-ten feature list of eight ML models.

Appendix Table 10 shows the top-five ranked feature list of each model with features 33ms_Fx (N) and 33ms_Fz (N) in the first ranking position. Each model emphasized different aspects of the dataset, showcasing diverse feature selection tendencies across machine learning algorithms. However, the features 33ms_Fx (N) and 33ms_Fz (N) appeared consistently across all models.

**Table 4:** Frequency of Features Appearing in the Top 10 Lists Across Models for BARBD Dataset.

Feature	Frequency
33ms_Fx (N)	8
33ms_My (N-m)	6
IC_My (N-m)	6
33ms_Fz (N)	6
IC_Fy (N)	6
67ms_My (N-m)	5
Sex	4
33ms_Fy (N)	3
MAX_Fy (N)	3
MIN_Fy (N)	3
IC_Mz (N-m)	3
MAX_ACL Linear Strain (%)	3
MAX_Fz (N)	2
67ms_Fz (N)	2
MIN_My (N-m)	2
IC_Fz (N)	2
MAX_Mz (N-m)	2
MIN_Fz (N)	2
MIN_ACL Linear Strain (%)	1
33ms_ACL Linear Strain (%)	1
33ms_Mz (N-m)	1
33ms_ACL Poly (mm)	1
67ms_Fx (N)	1
Height (cm)	1
MIN_Fx (N)	1
IC_Fx (N)	1
MAX_Mx (N-m)	1
MIN_Mz (N-m)	1
33ms_Mx (N-m)	1
MIN_ACL Linear (mm)	1
MAX_Fx (N)	1
33ms_ACL Linear (mm)	1
IC_Fz (N)	1

### ARW dataset

**Fig. 2:**
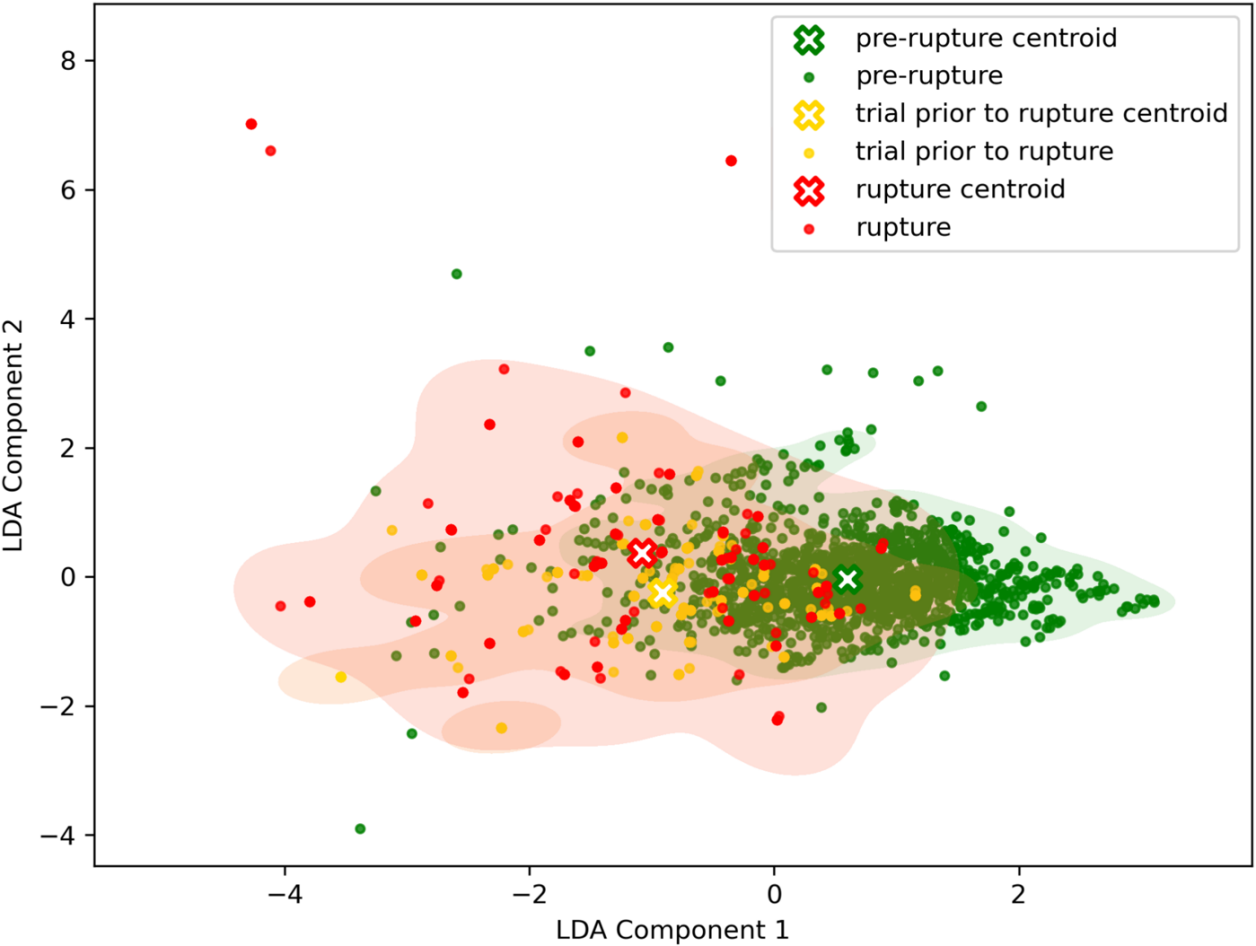
LDA of ARW dataset.

Table 5 summarizes the performance achieved by each model in the test set. The ARW dataset showed its highest accuracy of 76.5% using the SGD model, while most models had F1-macro scores in the low 50s. The results of the LDA plot showing reduced feature space for the ARW dataset can be observed in Fig. 2. There is a greater degree of overlap among cluster samples in Fig. 1, which corresponds to the ARBD dataset, compared to Fig. 2, which represents the ARW dataset. However, it can be said that in Fig. 2 the “trial prior to rupture” and “rupture” classes are leaning toward the left side of the plot, and samples of the “pre-rupture” class appear to be slightly toward the right side of the plot.

**Table 5:** Classification performance of ML models on ARW dataset.

Dataset	Model	Acc (%)	$F1_{ma}$(%)	$P_{ma}$(%)	$R_{ma}$(%)
ARW	LDA	70.6	51.8	54.5	51.4
	Ridge Regression	73.4	53.0	60.3	52.8
	Logistic Regression	72.3	52.3	57.8	52.3
	Gradient Boosting	71.7	48.9	57.6	48.6
	SVM	59.7	53.3	53.7	53.7
	Random Forest	69.1	41.9	40.9	44.2
	Decision Tree	65.8	52.7	54.1	51.7
	SGD	76.5	52.9	49.1	57.4
BARW	LDA	70.6	51.2	54.5	51.4
	Ridge Regression	81.5	79.2	79.9	78.7
	Logistic Regression	83.4	81.7	81.9	81.5
	Gradient Boosting	73.4	71.6	71.3	72.5
	SVM	78.9	77.3	76.8	78.0
	Random Forest	76.7	71.4	76.5	70.0
	Decision Tree	60.8	58.0	57.9	58.3
	SGD	81.7	79.4	80.2	78.8

ARW: ACL Rupture Wearable.

BARW: Binary ACL Rupture Wearable.

SVM: Support Vector Machine.

LDA: Linear Discriminant Analysis.

SGD: Stochastic Gradient Descent.

Table 6 demonstrates the frequency of appearance for each feature in the top-ten ranked feature lists across all models. IC_Fx (N) and IC_Fz (N) appeared in all eight models.

Appendix Table 11 demonstrates the top-five of ranked feature lists for each model based on the combined training and testing data sets. Sex and IC_Fy (N) appeared in the first rank position of the ranked feature lists of each model.

**Table 6:** Frequency of Features Appearing in the Top 5 Lists Across Models for ARW dataset.

Feature	Frequency
IC_Fx (N)	8
IC_Fz (N)	8
IC_Fy (N)	6
IC_My (N-m)	5
Sex	5
IC_ACL Linear Strain (%)	3
IC_Mz (N-m)	3
Race	1
IC_Mx (N-m)	1

### BARW dataset

The BARW dataset is similar to the ARW dataset, but ”trial prior to rupture” class and ”rupture” class are merged into one class. Table 5 summarizes the performance achieved by each model in the test set of the BARW dataset. Logistic Regression achieved the highest accuracy of 83.4% and F1-macro score of 81.7%, with several models reaching F1-macro scores above 70%. Same as the BARBD dataset, due to the binary nature of classes, an LDA plot could not be generated.

Table 7 shows a frequency table of the top-ten feature lists of each model. IC_Fx (N) and IC_Fz (N) appeared in all eight ML models’ top-ten ranked feature lists.

Appendix Table 12 demonstrates the top-five ranked features for each model using the merged training and testing datasets. The top-ranked feature of each model is identical with ARW dataset.

**Table 7:** Frequency of Features Appearing in the Top 5 Lists Across Models for BARW Dataset.

Feature	Frequency
IC_Fx (N)	8
IC_Fz (N)	8
IC_Fy (N)	6
Sex	6
IC_My (N-m)	4
IC_Mz (N-m)	3
IC_Mx (N-m)	2
Age	1
Height (cm)	1
IC_ACL Linear Strain (%)	1

## Discussion

Across ARBD/BARBD (high-dimensional laboratory biomechanical data) and ARW/BARW (13-feature wearable data), temporal impact metrics—33ms_Fx, 33ms_Fz, 67ms_My—and peak moment MAX_Mx consistently ranked highest, indicating strong positive correlation with imminent ACL failure. Demographics (Sex, Height) were secondary and dataset dependent. Notably, “trial prior to rupture” and “rupture” correlate closely, reducing separability; merging them into an “elevated risk” class increases model robustness given limited cadaver-derived samples and aligns better with clinical prevention priorities globally.

The primary observation from our results is the difference in performance between binary classification and three-class classification. Specifically, the performance of the binary classification model with BARBD and BARW datasets is notably higher than that of the three-class model with ARBD and BARW datasets. As noted in the methodology section, determining the presence of rupture during the data collection process was not straightforward for certain specimens. Consequently, there is an unintentional degree of uncertainty associated with the classification of some specimens into either class one or class two. Our findings demonstrate that “trial prior to rupture” class and “rupture” class exhibit lower specificity and sensitivity compared to “pre-rupture” class.

Based on observations of the results, the primary misclassifications occurred between “trial prior to rupture” class and “rupture” class. Statistical analysis supports this trend, showing a higher degree of data similarity and correlation between “trial prior to rupture” class and “rupture” class than between “pre-rupture” class and either of the other two classes. These results drives two key implications. First, distinguishing between “trial prior to rupture” class and “rupture” class presents a greater challenge for ML models than differentiating “pre-rupture” class from the other classes. Second, identifying the precise moment of rupture is more difficult than classifying a combination of ”rupture” and a sample ”trial prior to rupture” as an ”elevated risk” class compared to class zero. In addition, the class imbalance within the dataset, the smaller number of samples in “trial prior to rupture” class and “rupture” class relative to “pre-rupture” class, along with the limited dataset size, further contributes to the reduced specificity and sensitivity observed in the classification of “trial prior to rupture” class and “rupture” class.

According to Fig. 1, “trial prior to rupture” class and “rupture” class display similar behavioral patterns, which indicates a consistent development following the transition from the “pre-rupture” class. The data reveals a distinct and abrupt shift from “pre-rupture” class, which aligns with the initiation of injury. If “trial prior to rupture” class represents an initial or partial failure, the subsequent decline associated with “rupture” class signifies the completion of the failure process. This sequence of events reinforces the interpretation of ACL trauma as a sudden and acute injury, rather than a gradual deterioration^25^. By merging “trial prior to rupture” class and “rupture” class into ”elevated risk” class, the performance of ML models improved significantly. This reclassification not only enhances model performance but also aligns better with real-world applications by offering a more precise prediction of ACL rupture.

### Statistical analyses

Prior to performing ML techniques, ANOVA and t-test analyses were performed to provide insights on nuances between features that contribute to ACL injury. The results of these analyses show that certain features are indeed statistically different enough such that additional testing with ML models is encouraged to identify relevant features. From the results of the statistical analyses, we can see that between “pre-rupture” class and “trial prior to rupture” class, and “pre-rupture” class and “rupture” class, 48 and 46 features showed statistically significant difference, respectively. There is a noticeable drop in the number of significant features between “trial prior to rupture” class and “rupture” class, to only 19 features. Due to the drop in number of features statistically different between “trial prior to rupture” class and “rupture” class, it could be concluded that both these classes present with similar biomechanical signatures. At the point prior to rupture, the ACL is likely already exhibiting a similar biomechanic profile to the biomechanic profile of the ACL at the point of rupture. The statistical analysis results served as the basis for further exploration with ML to learn more on subtle differences that may exist between “trial prior to rupture” class and “rupture” class. The results of the statistical analyses supported the idea of merging “trial prior to rupture” class and “rupture” class, prompting additional ML experiments to further examine.

This observation prompts further testing to explore the nuances between “trial prior to rupture” class and “rupture” class that may contribute to ACL injury.

### ARBD dataset

The analysis of the ARBD dataset revealed behavioral patterns among the eight models. From Table 2, LDA achieved 86.9% accuracy and a 79.7% macro F1-score, closely followed by Ridge Regression with 85.6% accuracy and 78.0% F1-score, likely due to that when rich, high-dimensional biomechanical measures are available, linear projections can effectively separate pre-rupture, imminent rupture, and rupture states, perhaps by capturing global shifts in strain distribution and joint kinetics. Looking at Table 9, these linear models shared three common top features: MIN_ACL Linear, IC_ACL Linear, and 33ms_Fx, revealing their reliance on minimum strain values and early-phase forces.

Tree-based models (Gradient Boosting, Random Forest, Decision Tree) from Appendix Table 9 formed a distinct cluster prioritizing temporal force components, particularly 33ms_Fx and 33ms_Fz, with Gradient Boosting uniquely emphasizing IC_Fy. The SVM and SGD models bridged these groups, sharing MAX_Mx with Ridge Regression while also aligning with tree-based models through 33ms_Fz. Notably, SGD uniquely incorporated the demographic feature Sex, highlighting its sensitivity to demographic data.

In Table 3, MAX_Mx (N-m) and 33ms_Fx (N) each appear in six out of eight models’ top-10 lists, indicating strong consensus that peak knee moments and rapid anterior-posterior forces are key. These features represent the initial impact loading on the knee. This aligns with biomechanical findings that posterior ground reaction forces and knee extension moments strongly drive ACL loading^26^, as reported that peak ACL stress during landing correlates positively with posterior Ground-reaction forces and knee extension torque.

### BARBD datset

By merging the “trial prior to rupture” and “rupture” classes into a single “elevated risk” category, the BARBD dataset simplified the classification task to a binary problem. Model accuracy improved markedly into the mid-90% range parallels that of Kokkotis et al., who achieved $\sim$95% accuracy classifying ACL vs healthy using full gait kinetics^27^. This uniform boost across both linear and tree-based methods indicates that class consolidation reduces label ambiguity and enhances separability. Notably from Appendix Table 10, 33ms_Fx and 33ms_Fz were selected as the top feature by all eight models, and 33ms_My and IC_My each featured prominently in over half of the models’ top five lists. Features at or near initial contact (e.g. IC_Fx, IC_Fz, 33ms_Fx, MAX_Mx) appear repeatedly. This aligns with biomechanics: ACL strain peaks rapidly after landing (typically 50–60 ms post-contact^28^), so forces at 33 ms and 67 ms coincide with the critical injury window. The disappearance of demographic variables like sex and height from the highest-ranking positions implies that, under a binary framework, instantaneous biomechanical signatures dominate over subject-level covariates.

It can be seen in Appendix Table 10 that linear models (Ridge, Logistic) and SGD formed a cohesive group emphasizing temporal force components (33ms_Fx, 33ms_Fz), while tree-based models (Gradient Boosting, Random Forest, Decision Tree) created a distinct cluster focused on moment metrics (33ms_My, 67ms_My). The SVM model uniquely incorporated MAX_Fz, demonstrates different kernel-space representations. Notably, demographic features disappeared from top selections, indicating class merging reduced reliance on specimens characteristics.

From Table 4, the increase in 33ms_Fx frequency from 6 to 8 models and emergence of 67ms_My (N-m) as a key temporal marker -appearing in 5 models- indicate that simplified class structures amplify the importance of mid-phase biomechanical measurements. This convergence implies that time-specific force and moment metrics become increasingly discriminative when classifying binary.

### ARW dataset

The ARW dataset with 13 wearable features and three classes yielded generally lower accuracies based on Table 5-best SGD 76.5%, lowest SVM 60%, mirroring Crowe et al. (2020), who reported $\sim$73% accuracy using IMU sensors to distinguish post-ACL from controls in on-field tasks^29^. In fact, IC_Fx and IC_Fz appeared in all models of Table 11, indicating among wearable features, the first-foot-contact forces provide the strongest signal about ACL risk class. Three distinct behavioral groups emerged: (1) Demographic-sensitive models like Ridge, Logistic, and SVM, incorporating Sex. Female sex is a well-known risk factor—Taborri et al. note that female athletes have up to eight times higher ACL injury incidence than males^30^—and its presence in linear models which can directly use this demographic binary to boost their performance. (2) pure biomechanical models like Random Forest, Gradient Boosting, focusing on IC_Fy and IC_Mz encode the actual load, (3) hybrid models (SGD, Decision Tree) blending strain metrics with force components.

Notably from Appendix Table 11, the LDA model uniquely prioritized Race, while other models ignored demographic factors except Sex. The universal reliance on IC metrics, appearing more frequently than in ARBD dataset analyses, confirms wearable systems’ dependency on early-phase movement characteristics. Despite lower overall accuracy compared to the ARBD dataset, the strong consensus on IC features indicate that wearable-based systems require precise measurement of initial ground contact forces.

### BARW dataset

The binary BARW dataset showed very similar feature importance to ARW, with IC_Fx and IC_Fz appearing universally as shown in Appendix Table 12. Four behavioral patterns emerged: (1) Demographic-force hybrids (Ridge, Logistic, SVM) combining Sex with IC forces, (2) Moment-focused models (Random Forest, Gradient Boosting) emphasizing IC_Mz/Mx, (3) Strain-integrating models (SGD, Decision Tree) incorporating ACL Linear Strain, (4) SVM’s top-5 included Height and Decision Tree included Age, revealing these anthropometric factors have minor predictive value when classes are merged.

Notably from Table 5, merging classes improved SGD performance by 5.2% while degrading Decision Tree accuracy by 4.0%, indicating that tree-based methods struggle with reduced class complexity in ARW data.

### Limitation and future work

One limitation of this study is that it focused solely on a classification problem and was restricted to two or three classes. This constraint may limit the comprehensiveness of the information, particularly in the context of risk profiling. We aim to expand our approach by incorporating a broader range of risk levels, which will provide more detailed and informative insights for users. This could be accomplished by stratifying a spectrum of risk factors to each specimen based on their demographic information. Secondly, this study is limited by a relatively small sample size for ML models. The current dataset was derived exclusively from cadaveric samples; as such, generation of a population comprehensive dataset is unrealistic due to resouce demands. Accordingly, the development of a wearable solution for subsequent *in vivo* field implementation is necessary.

Future work relative to this analysis will concentrate on the development of a wearable device for real-time biofeedback during *in vivo* athletics participation and injury rehabilitation. Successful integration of the ML model differentiation process presented in this study into real-world participant risk-identification would be revolutionary to preventive biomechanics care of athletes and active individuals. This will substantially expand the dataset and introduce real-world failure events to more precisely tune the ML models. Further, incorporation of additional data sources, such as Inertial Measurement Unit (IMU) signals, will enable capture of dynamic, real-world movement patterns. With a more comprehensive dataset, we plan to explore deep learning algorithms on both raw and newly collected data, aiming to enhance predictive accuracy and model robustness. in general, deep learning algorithms require more data to reach their full capacity.

Furthermore, the methodological framework presented in this study is not confined to ACL injury prediction. Future research may extend these approaches to other injury types, such as those affecting additional joint across the body, thereby broadening the clinical applicability of our work.

## Conclusion

This study demonstrates the viability of ML models for extracting and ranking biomechanical features critical to predicting ACL injuries. Our analysis, which encompassed a diverse set of models, including linear techniques (Ridge and Logistic Regression), ensemble methods (Random Forest and Gradient Boosting), and dimensionality reduction via linear discriminant analysis, revealed a robust consensus on key predictors. In particular, early-phase force metrics (e.g., 33ms_Fx and 33ms_Fz) consistently emerged as significant across multiple models, underscoring their potential as early warning indicators for the risk of ACL injury.

Furthermore, the reclassification strategy, merging closely related classes into a binary schema (“pre-rupture” vs. “elevated risk”), yielded substantial improvements in model accuracy and generalizability. This not only aligns with the clinical notion that ACL injuries are a sudden and acute injury but also enhances the practical applicability of real-time monitoring scenarios. The incorporation of wearable sensor data, despite inherent challenges related to data noise and limited feature availability, reinforced the central role of initial contact metrics and highlighted the promise of deploying such systems in dynamic real-life settings.

The integration of advanced feature extraction methods with robust machine learning algorithms contributes a significant step forward in ACL injury prediction. These results provide a foundation for future work aimed at real-time injury prevention and personalized rehabilitation protocols. Future research should focus on expanding data collection, exploring deep learning techniques, and integrating additional injury modalities to further enhance predictive capabilities and clinical utility.

## Data Availability

The data that support the findings of this study are available from the Director of the University of South Florida - Center of Neuromusculoskeletal Research, [N.D. Schilaty], upon reasonable request.

## Appendix

See Tables 8,9,10,11,12 and 13.

**Table 8:** Feature list.

Feature	Description
side*	L or R limb
MAX_Fx (N)	Max force in X
MAX_Fy (N)	Max force in Y
MAX_Fz (N)	Max force in Z
MAX_Mx (N-m)	Max moment in X
MAX_My (N-m)	Max moment in Y
MAX_Mz (N-m)	Max moment in Z
MAX_ACL Linear (mm)	Maximum ACL displacement (mm) calculated with linear equation
MAX_ACL Poly (mm)	Maximum ACL displacement (mm) calculated with polynomial equation
MAX_ACL Linear Strain (%)	Maximum ACL strain (%) [mm/mm]
MIN_Fx (N)	Min force in X
MIN_Fy (N)	Min force in Y
MIN_Fz (N)	Min force in Z
MIN_Mx (N-m)	Min moment in X
MIN_My (N-m)	Min moment in Y
MIN_Mz (N-m)	Min moment in Z
MIN_ACL Linear (mm)	Minimum ACL displacement (mm) calculated with linear equation
MIN_ACL Poly (mm)	Minimum ACL displacement (mm) calculated with polynomial equation
MIN_ACL Linear Strain (%)	Minimum ACL strain (%) [mm/mm]
IC_Fx (N)*	Force in X at initial contact
IC_Fy (N)*	Force in Y at initial contact
IC_Fz (N)*	Force in Z at initial contact
IC_Mx (N-m)*	Moment in X at initial contact
IC_My (N-m)*	Moment in Y at initial contact
IC_Mz (N-m)*	Moment in Z at initial contact
IC_ACL Linear (mm)	ACL displacement (mm) at initial contact calculated with linear equation
IC_ACL Poly (mm)	ACL displacement (mm) at initial contact calculated with polynomial equation
IC_ACL Linear Strain (%)*	ACL strain (%) at initial contact [mm/mm]
33ms_Fx (N)	Force in X at 33 msec after initial contact
33ms_Fy (N)	Force in Y at 33 msec after initial contact
33ms_Fz (N)	Force in Z at 33 msec after initial contact
33ms_Mx (N-m)	Moment in X at 33 msec after initial contact
33ms_My (N-m)	Moment in Y at 33 msec after initial contact
33ms_Mz (N-m)	Moment in Z at 33 msec after initial contact
33ms_ACL Linear (mm)	ACL displacement (mm) at 33 msec after initial contact calculated with linear equation
33ms_ACL Poly (mm)	ACL displacement (mm) at 33 msec after initial contact calculated with polynomial equation
33ms_ACL Linear Strain (%)	ACL strain (%) at 33 msec after initial contact [mm/mm]
67ms_Fx (N)	Force in X at 66 msec after initial contact
67ms_Fy (N)	Force in Y at 66 msec after initial contact
67ms_Fz (N)	Force in Z at 66 msec after initial contact
67ms_Mx (N-m)	Moment in X at 66 msec after initial contact
67ms_My (N-m)	Moment in Y at 66 msec after initial contact
67ms_Mz (N-m)	Moment in Z at 66 msec after initial contact
67ms_ACL Linear (mm)	Force at 66 msec after initial contact on externally applied load
67ms_ACL Poly (mm)	Force at 66 msec after initial contact on externally applied load
67ms_ACL Linear Strain (%)	Force at 66 msec after initial contact on externally applied load
ACL DVRT voltage baseline	Calibrated neutral voltage of DVRT. When this value is zero, there is no displacement of the ACL.
Mass (Kg)	Full body weight of donor at death
Age*	Age in years of specimen at death
BMI*	Body mass index
Height (cm)*	Height of donor at death
Sex*	Sex of donor
Race*	Race of donor

$^{*}$ These features are present only in ACL Rupture Wearable (ARW), and Binary ACL Rupture Wearable (BARW) datasets.

**Table 9:** Top 5 Ranked Features of ARBD Dataset.

Model	Top 5 ranked feature list				
Ridge Regression	MIN_ACL Linear (mm)	IC_ACL Linear (mm)	33ms_Fz (N)	MAX_Mx (N-m)	33ms_Fx (N)
Logistic Regression	IC_ACL Linear (mm)	MIN_ACL Linear (mm)	MAX_ACL Linear (mm)	33ms_ACL Linear (mm)	MAX_Mz (N-m)
Gradient Boosting	33ms_Fx (N)	33ms_My (N-m)	MAX_Fy (N)	IC_Fy (N)	MIN_Fx (N)
SVM	MIN_Fx (N)	MAX_Mx (N-m)	33ms_Fz (N)	MAX_My (N-m)	MAX_Fy (N)
Random Forest	33ms_Fx (N)	33ms_Fz (N)	33ms_My (N-m)	67ms_Fz (N)	IC_Fy (N)
Decision Tree	33ms_Fx (N)	MAX_Fy (N)	IC_Fy (N)	MIN_Fz (N)	MAX_ACL Linear Strain (%)
SGD	MAX_Mx (N-m)	67ms_ACL Poly (mm)	33ms_Fz (N)	Sex	MAX_Mz (N-m)
LDA	33ms_Fz (N)	33ms_Fx (N)	MIN_Fz (N)	IC_My (N-m)	MAX_Mx (N-m)

**Table 10:** Top 5 Ranked Features of BARBD Dataset.

Model	Top 5 ranked feature list				
Ridge Regression	33ms_Fz (N)	33ms_Fx (N)	MIN_Fz (N)	IC_My (N-m)	MIN_ACL Linear (mm)
Logistic Regression	33ms_Fx (N)	33ms_Fz (N)	33ms_My (N-m)	67ms_My (N-m)	67ms_Fz (N)
Gradient Boosting	33ms_Fx (N)	33ms_My (N-m)	IC_Fy (N)	IC_My (N-m)	67ms_My (N-m)
SVM	33ms_Fz (N)	33ms_Fx (N)	IC_My (N-m)	MIN_Fy (N)	MAX_Fz (N)
Random Forest	33ms_Fx (N)	33ms_Fz (N)	33ms_My (N-m)	67ms_My (N-m)	67ms_Fz (N)
Decision Tree	33ms_Fx (N)	IC_Fy (N)	33ms_My (N-m)	67ms_My (N-m)	MAX_ACL Linear Strain (%)
SGD	33ms_Fz (N)	33ms_Fx (N)	33ms_My (N-m)	IC_My (N-m)	MIN_Fy (N)
LDA	33ms_Fz (N)	33ms_Fx (N)	MIN_Fz (N)	IC_My (N-m)	IC_Mz (N-m)

**Table 11:** Top 5 Ranked Features of ARW Dataset.

Model	Top 5 ranked features				
Ridge Regression	Sex	IC_My (N-m)	IC_Fy (N)	IC_Fx (N)	IC_Fz (N)
Logistic Regression	Sex	IC_Fy (N)	IC_Fx (N)	IC_My (N-m)	IC_Fz (N)
Gradient Boosting	IC_Fy (N)	IC_Fz (N)	IC_Fx (N)	Sex	IC_Mz (N-m)
SVM	Sex	IC_Fz (N)	IC_Fx (N)	IC_My (N-m)	Race
Random Forest	IC_Fy (N)	IC_Fz (N)	IC_Fx (N)	IC_ACL Linear Strain (%)	IC_Mz (N-m)
Decision Tree	IC_Fy (N)	IC_Fz (N)	IC_My (N-m)	IC_Fx (N)	IC_ACL Linear Strain (%)
SGD	IC_Fy (N)	IC_Fx (N)	IC_Mx (N-m)	IC_Fz (N)	IC_ACL Linear Strain (%)
LDA	Sex	IC_Fx (N)	IC_My (N-m)	IC_Fz (N)	IC_Mz (N-m)

**Table 12:** Top 5 Ranked Features of BARW Dataset.

Model	Top 5 ranked feature list				
Ridge Regression	Sex	IC_My (N-m)	IC_Fy (N)	IC_Fx (N)	IC_Fz (N)
Logistic Regression	Sex	IC_Fy (N)	IC_Fx (N)	IC_My (N-m)	IC_Fz (N)
Gradient Boosting	IC_Fy (N)	IC_Fz (N)	IC_Fx (N)	Sex	IC_Mz (N-m)
SVM	Sex	IC_Fz (N)	IC_Fx (N)	IC_My (N-m)	Height (in)
Random Forest	IC_Fy (N)	IC_Fz (N)	IC_Fx (N)	IC_Mz (N-m)	IC_Mx (N-m)
Decision Tree	IC_Fy (N)	IC_Fx (N)	IC_Fz (N)	Sex	Age
SGD	IC_Fy (N)	IC_Fx (N)	IC_Mx (N-m)	IC_Fz (N)	IC_ACL Linear Strain (%)
LDA	Sex	IC_Fx (N)	IC_My (N-m)	IC_Fz (N)	IC_Mz (N-m)

**Table 13:** List of Abbreviations and Definitions.

Abbreviation	Definition
ACL	Anterior Cruciate Ligament.
ACLR	Anterior Cruciate Ligament reconstruction.
Acc	Accuracy (ratio of correctly classified samples).
ARBD	ACL Rupture Biomechanical and Demographic dataset.
ARW	ACL Rupture Wearable dataset.
ATT	Anterior Tibial Translation.
BARBD	Binary ACL Rupture Biomechanical and Demographic dataset.
BARW	Binary ACL Rupture Wearable dataset.
DT	Decision Tree classifier.
DVRT	Differential Variable Reluctance Transducer (sensor measuring ligament displacement).
$F1_{ma}$	Macro-averaged F1-score.
GB	Gradient Boosting.
IMU	Inertial Measurement Unit (accelerometer + gyroscope).
ITR	Internal Tibial Rotation.
KAM	Knee Abduction Moment.
LDA	Linear Discriminant Analysis.
LR	Logistic Regression.
MCL	Medial Collateral Ligament.
MRI	Magnetic Resonance Imaging.
mm	Millimeter (unit of displacement).
N	Newton (unit of force).
N-m	Newton-meter (unit of moment).
$P_{ma}$	Macro-averaged Precision.
RF	Random Forest.
$R_{ma}$	Macro-averaged Recall.
Ridge	Ridge Regression (L2-regularized linear model).
SGD	Stochastic Gradient Descent classifier.
SVM	Support Vector Machine.
